# State Medical Association Meeting at Augusta

**Published:** 1896-05

**Authors:** 


### Editorial.

### STATE MEDICAL ASSOCIATION'S MEETING AT AUGUSTA.

The Medical Association of Georgia met at Augusta, April 15, 16, 17. The address of welcome was gracefully delivered by Eugene Foster, of Augusta. In response Dr. E. R. Anthony, of Griffin, called attention to the appropriateness of the meeting taking place in Augusta, for there repose the bones of some of the most celebrated workers in the field of medicine that the state has produced, Henry F. Campbell, the Eves and Dugas. The city of Augusta should be to the medical profession of Georgia as Mecca to the Mohammedan.

Dr. F. M. Ridley delivered the president's address, in which was displayed his well-known ability as an orator.

On the afternoon of the second day the Association adjourned to partake of the barbecue so generously provided by the physicians at the Schuetzen Platz. It combined not only the features of a barbecue, but those of an elaborate banquet of which the members of the Association showed their appreciation in a most substantial manner. In the evening the members in attendance were treated to a soiree musicale given to their entertainment.

The election of officers for the next year on the third day resulted in Dr. George H. Noble, of Atlanta, President, Dr. J. B. Morgan, of Augusta, Vice-President, Dr. R. B. Barron, of Macon, second Vice-President. Atlanta is to be congratulated in being represented again so ably in the president's chair.

The meeting was a very delightful one and much credit and thanks are due to the physicians of Augusta for the success of their efforts at entertainment and hospitable kindness. The meeting adjourned on the 17th, to meet in Macon next year.

